# Mechanical Behavior of 3D-Printed Zig-Zag Honeycomb Structures Made of BASF Ultrafuse 316L

**DOI:** 10.3390/ma17246194

**Published:** 2024-12-18

**Authors:** Marcin Sarzyński, Paweł Płatek, Patryk Cedro, Urvashi Gunputh, Paul Wood, Alexis Rusinek

**Affiliations:** 1 Faculty of Mechatronics, Armament and Aerospace, Military University of Technology, 2 Gen. S. Kaliskiego Street, 00-908 Warsaw, Poland; pawel.platek@wat.edu.pl (P.P.); patryk.cedro@student.wat.edu.pl (P.C.); 2 Institute for Innovation in Sustainable Engineering, College of Science and Engineering, University of Derby, Kedleston Rd., Derby DE22 1GB, UK; u.gunputh@derby.ac.uk (U.G.); p.wood7@derby.ac.uk (P.W.); 3 Laboratory of Microstructure Studies and Mechanics of Materials (LEM3), Lorraine University, UMR CNRS 7239, 57078 Metz, France; alexis.rusinek@univ-lorraine.fr

**Keywords:** 316L, additive manufacturing, material extrusion, FDM, quasi-static crushing test, energy absorption, honeycomb, cellular structures

## Abstract

The aim of this study is to determine the mechanical behavior of 2D honeycomb cellular structures with deformation initiators subject to quasi-static compression testing. Two different loading directions were studied: in-plane (IP) and out-of-plane (OP). The deformation initiators sought to stabilize the mechanical response by decreasing the initial peak force in the case of OP loading. The samples for testing were made using stainless steel 316L that was 3D-printed using material extrusion (MEX). The method enables fabrication of structures with high mechanical strength and ductility. The findings of the quasi-static compression testing showed that the additional deformation initiators were able to significantly reduce the orthotropy in the mechanical response of honeycomb cellular structures.

## 1. Introduction

Additive manufacturing (AM) techniques are among the topics most frequently discussed in mechanical engineering and other manufacturing technologies [1]. Among all additive manufacturing techniques, one of the most commonly used is FDM/FFF (filament deposition modelling/fused filament fabrication), due to its many advantages such as accessibility, simplicity of the 3D printing process, and low cost of both equipment and materials [2]. According to the ISO/ASTM 52900:2021 standard, this technique is referred to as material extrusion (MEX). Its definition is “a process in which material is selectively dispensed through a nozzle or orifice” [3]. The key issue of this process is related to the melting process of polymeric materials carried out in the extruder nozzle and subsequent deposited on the working bed ”layer by layer”.

Analyzing the potential of additive manufacturing in the field of metal part production, one type of material that increasingly being used is a mixture of polymers with metal powders [4]. This technique is commonly called metal FDM, which is a relatively new method based on the standard FDM process [5]. In metal FDM, the equipment used for producing elements with metal composites is similar to that used for the polymer 3D printing process. Instead of standard brass nozzles, dedicated heat-treated steel nozzles are used because of their greater hardness and wear resistance. After 3D printing process, it is necessary to perform the debinding process, which involves removing the binder matrix from the metal particles. The final stage is the catalytic sintering process, where the material is subjected to high temperature, resulting in melting and fusing of the powder particles. This process is also carried out in a gas atmosphere [6]. As a result of this process, the final components exhibit a uniform metallic structure [7]. Both during the debinding and sintering, the duration of the process is a significant parameter and affects the final microstructural properties of the obtained components [8]. The properties of materials produced using this technique are presented in [9,10].

Additive manufacturing techniques, compared to other conventional production methods, offer immense capabilities for complex structure fabrication [11]. Nazir et al. [12] presented the types of cellular structures most commonly manufactured using the FDM technique; in most cases, they were inspired by nature. In addition to structures directly derived from nature, generative design, often supported by artificial intelligence algorithms, offers significant design potential. Such structures, such as lattices or honeycombs, can be designed to minimize weight while maintaining high strength [13], which is highly demanded in various engineering applications such as aerospace [14], automotive [15], biomechanics [16], and applications related to dynamic load wave propagation [17]. Reduced component weight leads to lower fuel consumption and enables reductions in cost [18]. Furthermore, the nature of cellular structures allows for more efficient energy absorption, which can be used in various crashworthiness applications like energy-absorbing barriers, bumpers, and other safety components [19]. Furthermore, these structures exhibit good heat dissipation [20] and excellent compressive strength [21]. Furthermore, additive manufacturing techniques enable faster iterations in the design and production process [22].

One of the most popular manufactured regular cell structures is the “honeycomb”, based on a series of hexagons arranged side by side [23]. The use of additive manufacturing techniques enables the production of such structures in any configuration, according to the design defined based on the result of the numerical simulations. By applying the appropriate configurations during the design phase, it is possible to achieve structures with auxetic behavior [24]. In a comprehensive study by J. Zhang et al., the process of fabricating auxetic structures using both polymers and metals is presented [25]. The results of the simulations and experimental studies presented in the article were found to be similar.

In the context of research on energy absorption by regular cellular structures, additive manufacturing represents a key role, enabling the design and production of structures optimized for specific needs and loading conditions. When the capabilities and level of advancement of additive manufacturing techniques for metals are considered, this field is less explored than that of techniques involving polymers. The scientific literature primarily provides information on the production and study of cylindrical [26] and triangular [27] auxetic structures using the LPBF (laser powder bed fusion) 3D printing technique. Despite this, the available materials and publications reveal a limited amount of information on cellular structures with the geometry presented in this article, produced using the FDM technique with 316L steel.

The main objective–research problem of this study is to determine the mechanical response of 2D honeycomb cellular structures with deformation initiators under compression testing, considering two different loading directions: in-plane and out-of-plane. The aim of this work was to explore the possibility of reducing the peak force occurring in the out-of-plane loading direction. Using BASF Ultrafuse 316L, the samples were 3D printed using material extrusion (MEX). This approach enables 3D printing of 316L stainless steel honeycomb structures with high mechanical strength and ductility. Compared to commonly used techniques such as LPBF and DED, the MEX 3D printing offers lower production costs and does not require expensive AM equipment. The results obtained will serve as a starting point for further work involving computer simulations and examining the mechanical response of structures under dynamic loading conditions.

## 2. Materials and Methods

### 2.1. Development of Structure Topologies

**Figure 1:**
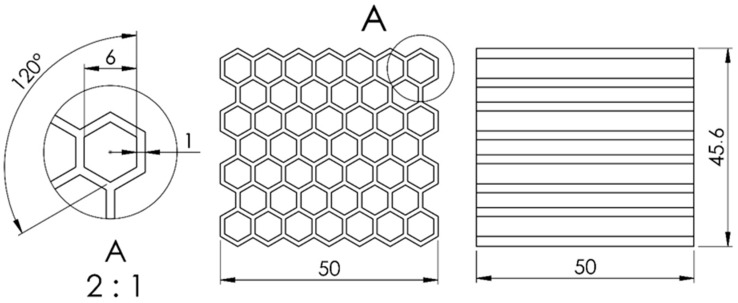
Dimensions of the unit cell (detail view A) and the entire structure.

A cross-section of a honeycomb structure as shown in Figure 1 was considered in this paper. The dimensions of a single unit cell common to all variants were as follows:

- Unit cell size—6 mm;
- Unit cell wall thickness—1 mm;
- Unit cell height—50 mm.

The honeycomb structure should consist of at least six unit cells in two directions in-plane [28] to avoid an unstable buckling collapse. In this study, the final size of the structure was seven unit cells in two directions in-plane to maintain symmetry. The overall dimensions of the honeycomb structure were 45.6 mm × 50 mm × 50 mm, with a total number of 46 unit cells, as shown in Figure 1.

The relative density of a single unit cell of a honeycomb structure with a regular hexagonal cross-section can be calculated using Formula (1):

(1) $$\rho_{r}=\frac{t\left(2R+t\right)}{{\left(R+t\right)}^{2}}=\frac{t\left(2l+t\right)}{{\left(l+t\right)}^{2}}$$

Parameters:

ρ_r_—relative density;

t—wall thickness of the cell;

R—radius of the circle inscribed in the cell;

l—cell size, where l = R.

According to (1), the relative density of a honeycomb structure designed with parameters t = 1 and R = 6 is approximately ρ_r_ = 0.27. This value was typical for the honeycomb structure described in [29,30,31].

**Figure 2:**
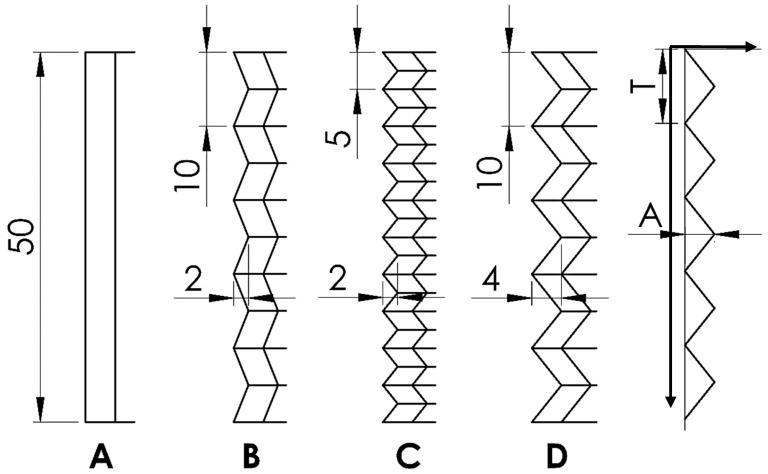
The longitudinal cross-sectional profiles of the designed cellular structures are shown from left to right as follows: (**A**) (straight profile), (**B**) (A = 2 mm, T = 10 mm), (**C**) (A = 2 mm, T = 5 mm), (**D**) (A = 4 mm, T = 10 mm). Geometry of each profile is described by the parameters of the periodic, single-sided, zero-offset triangular function.

**Figure 3:**
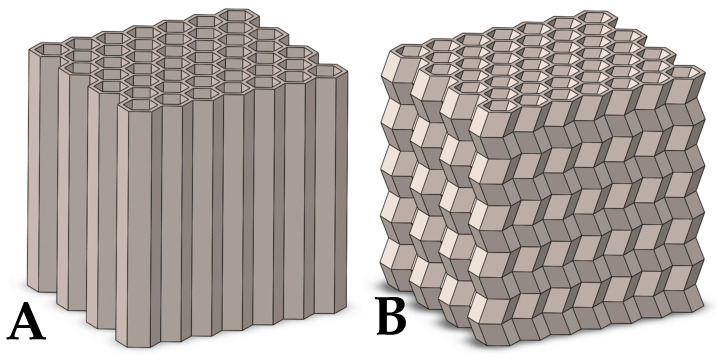

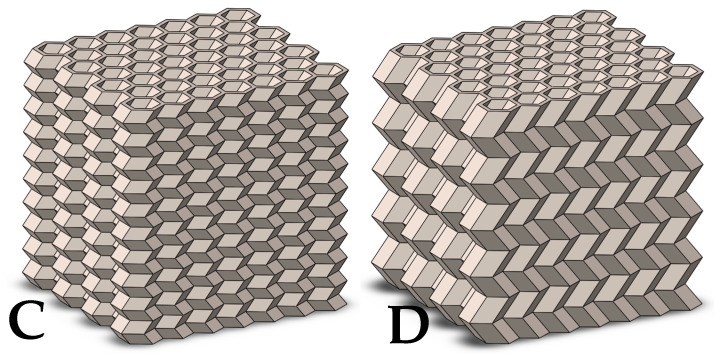
Geometric models of the designed cellular structures: (**A**) (straight profile), (**B**) (A = 2 mm, T = 10 mm), (**C**) (A = 2mm, T = 5mm), (**D**) (A = 4 mm, T = 10 mm).

Deformation initiators were introduced on the sidewalls that sought to stabilize the mechanical response of the honeycomb structure when subject to crush loading in two orthogonal loading directions [23]. The proposed variations of the deformation initiator geometries are shown in Figure 2. The geometrical features of these deformation initiators are a periodic zig-zag pattern described by two parameters: amplitude (A) and period (T). Four variants of the deformation initiators were considered in this study, labelled A to D, and are shown in Figure 2 and Figure 3. Type A is the standard honeycomb structure, whereas types B, C, and D are modified variants of the zigzag profiles. Figure 2 shows the longitudinal cross-sectional profiles and the parameters of the periodic, single-sided, zero-offset triangular function describing them.

Using SolidWorks v2022 CAD software (Dassault Systèmes, Vélizy-Villacoublay, France), geometric models were created for the variants described above. Figure 3 shows the models of the four types studied.

### 2.2. Characteristics of Implemented Material in the 3D Printing Process

The samples were produced using a metal–polymer composite filament, Ultrafuse 316L Stainless Steel, from BASF 3D Printing Solutions GmbH (Ludwigshafen, Germany). This material is a derivative of BASF’s Catamold^®^ (Heidelberg, Germany) product family that is used in metal injection molding (MIM). It enables the safe, simple, and cost-effective production of fully metallic part prototypes, metal tools, or functional metal components via a 3D printing process. After 3D printing, parts must undergo the standard MIM processes of debinding and sintering to achieve the appropriate mechanical properties. The filament features a non-slip surface, making it compatible with any Bowden or direct drive extruder used in FFF 3D printers. Despite the high metal powder content (more than 80% by mass), the filament is flexible and works well with the extruders of popular printers. Table 1 shows the mechanical parameters provided by the manufacturer (BASF 3D Printing Solutions GmbH) that were determined using the respective ISO standard methods.

### 2.3. Manufacturing Process

The honeycomb structure samples were produced using the Prusa i3 MK3S 3D printer (Prague, Czech Republic), which uses fused filament fabrication (FFF) technology. This process extrudes the thermoplastic filament as a melt. The build volume is 250 × 210 × 200 mm, with a horizontal printing accuracy on the 0XY plane of ±0.05 mm and a maximum printing speed of 200 mm/s. PrusaSlicer v2.5.2 software was used to prepare the G-code files. This slicing software is dedicated to Prusa 3D printers.

Using BASF Ultrafuse 316L metal filament, the following 3D printing parameters were used for all samples:

- Nozzle temperature: 250 °C—This is the upper limit of the recommended temperature range for this filament. The higher temperature reduces the risk of nozzle clogging and improves the adhesive bonding between layers.
- Build plate temperature: 100 °C—Heating the build plate ensures better adhesion of the first layer of the printed object to the working bed surface and prevents warping during printing.
- Cooling: Disabled—According to the filament manufacturer, any cooling during printing can cause warping and deformation due to the rapid, uncontrolled shrinkage of the material.
- Layer height: 0.15 mm—This value provides an optimal balance between geometric accuracy and required printing time.
- Height of the first layer: 0.2 mm—The increased height of the first layer improves adhesion to the build plate and compensates for working bed surface imperfections.
- Infill density: 100%—This recommended density ensures proper subsequent processing (polymer debinding and sintering).
- Brim: 10mm wide—An additional layer of material printed around the model base, increasing the model’s adherence to the build plate and potentially reducing distortions caused by material contraction, a common issue that arrives when metal filaments are used.

**Figure 4:**
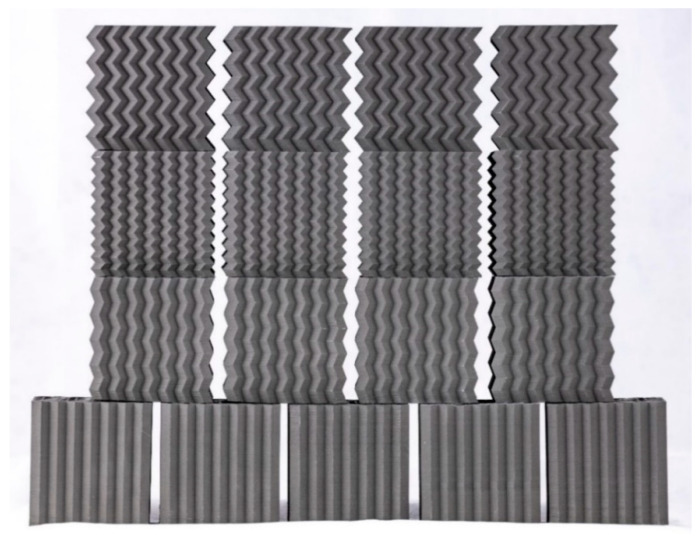
Structures printed with BASF Ultrafuse 316L metal filament (“green part”) before debinding and sintering.

To improve the adhesion of the prints on the build plate, Magigoo PRO Metal adhesive (Magigoo, Malta), as recommended by the BASF Ultrafuse filament manufacturer, was applied. This adhesive improves the bonding of the first layer of the print while allowing for easy and safe removal of finished parts from the build platform after cooling, without the need for tools. A total of 17 samples were produced: five in type A (straight longitudinal cross-section), and four each in types B, C, and D (with a longitudinal cross-section profile described by the triangular function). The green part samples are shown in Figure 4.

In the subsequent stage, the green parts were subjected to the debinding process to remove the polymer that binds the metal powder particles. This process was carried out using specialized equipment through a thermochemical catalytic reaction, where the heated parts are exposed to nitric acid vapors within a nitrogen atmosphere. Following this step, the so-called brown parts are produced.

**Figure 5:**
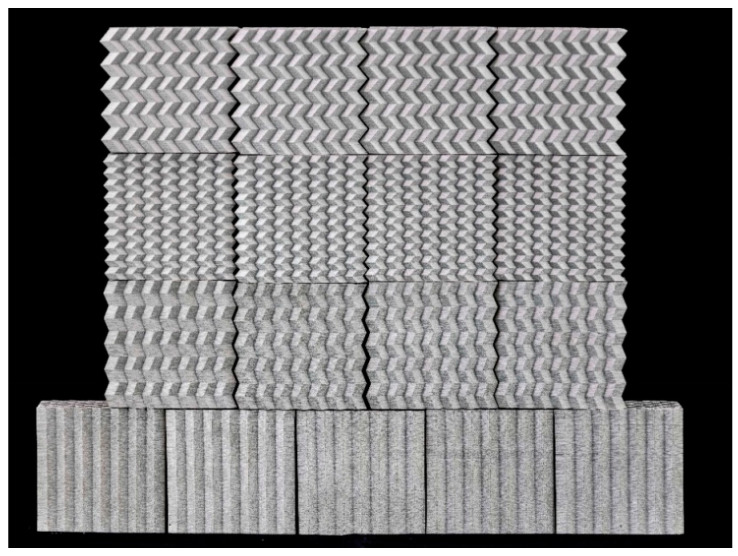
Cellular structures after debinding and sintering processes.

Immediately after the debinding process, the parts must be transferred to a furnace for final sintering at a high temperature (~1400 °C). The debinding and sintering of the Ultrafuse 316L parts was carried out by Elnik Systems GmbH (Ebhausen, Germany). The complete set of samples produced in this manner is shown in Figure 5.

**Figure 6:**
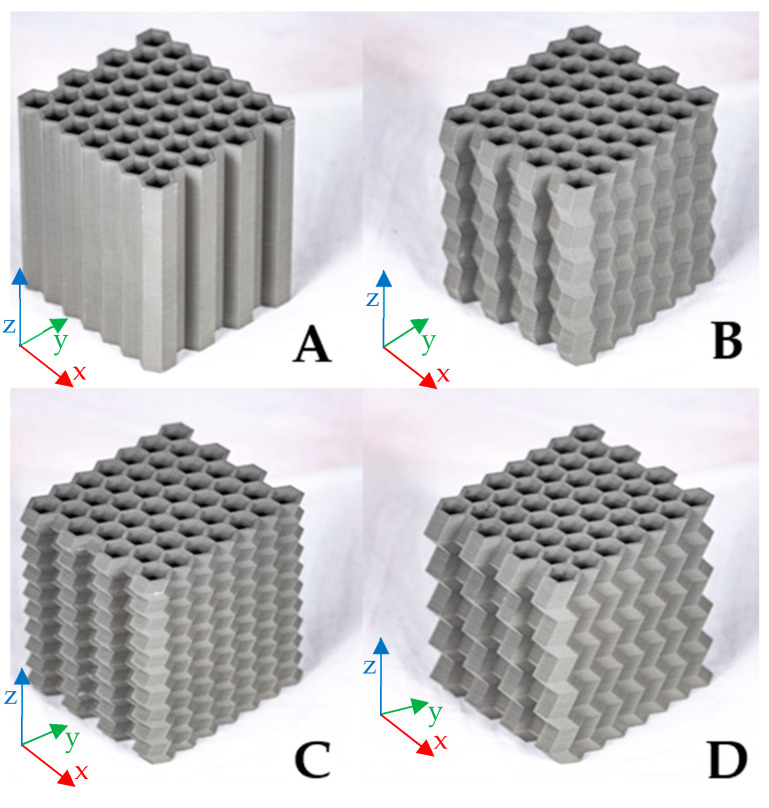
View of the four types (**A**–**D**) of cellular structures prepared for compression tests.

Figure 6 shows the view of each sample revealing the structure’s topology.

### 2.4. Quasi-Static Crushing Test of Structure Samples

The structures fabricated as described above were subjected to quasi-static crush tests. Eight (two for each type) out of the seventeen manufactured specimens were chosen for this analysis, while the remaining samples were reserved for subsequent dynamic loading experiments. Compression tests were performed in two orthogonal loading directions for each sample group. For clarity, the following designations were assigned to the test orientations of the samples:

- In-plane (IP) orientation: aligned with the X-axis of the CAD models, where the axis of the unit cell is perpendicular to the direction of the applied force;
- Out-of-plane (OP) orientation: aligned with the Z-axis of the CAD models, where the axis of the unit cell is parallel to the direction of the applied force.

**Figure 7:**
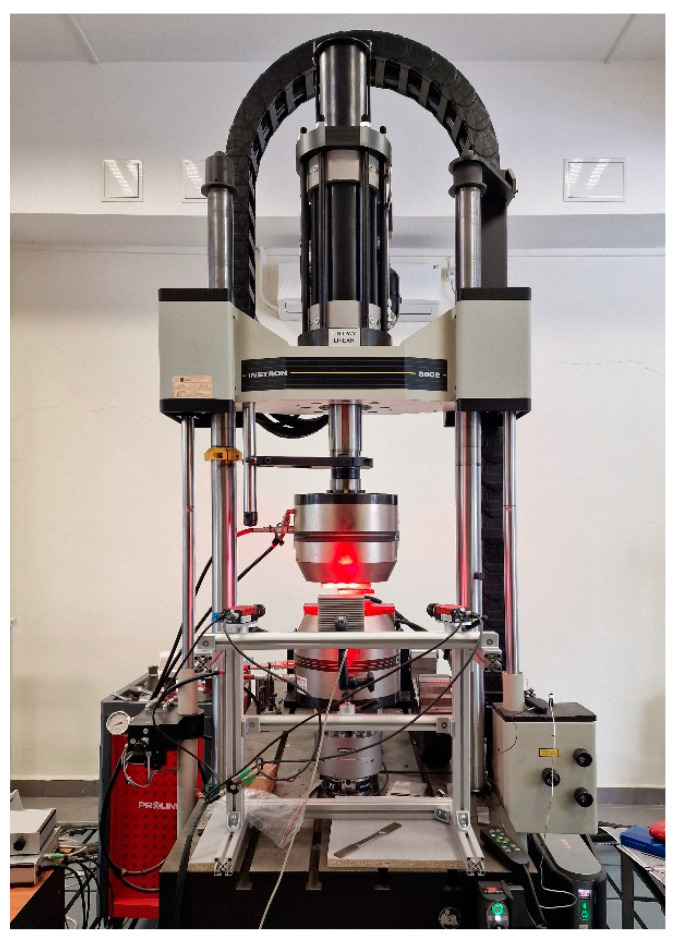
Servo-hydraulic universal testing system, Instron 8802 (Norwood, MA, USA).

The tests were performed using a universal servo-hydraulic testing system, Instron 8802 (Norwood, MA, USA)—Figure 7. The key parameters of the test included the following:

- Traverse speed: 0.1 mm/s;
- Data acquisition frequency: 10 Hz;
- Test termination condition: Achievement of ~200 kN load or 50% compression, with the possibility of early termination of the test if there was potential risk to the operator or equipment.

A crucial parameter in the design of energy-absorbing structures is their capacity to absorb plastic strain energy during deformation. This energy absorption is represented by the area under the force displacement history plot. A quantitative assessment of the absorbed energy can be obtained using Equation (2).

(2) ∆E=∆F·∆x

Parameters:

E—energy [J];

F—load [kN];

x—deformation [mm].

## 3. Results

### 3.1. Mass and Dimensional Properties of Structures Subjected to the Debinding and Sintering Process

The mass of each sample and the actual dimensions were measured before and after the debinding and catalytic sintering processes as shown in Table 2. The average percentage reduction in mass and dimensions after shrinkage for each group of samples were determined.

In all cases, a comparable reduction in the mass and dimensions of the samples was observed, which indicates a uniform contraction due to the debinding and catalytic sintering process. The largest mass reduction was achieved in type A (11.45%), while the smallest reductions were observed in type B and C (11.36%), but these differences are very small. The greatest width decrease was noted for type A (16.49%) and the smallest for type D (16.09%). The greatest length decrease was recorded for type D (16.78%), while the smallest was type A (16.23%). The most significant decrease in height was found in type D (22.08%) and the least in type A (20.76%).

**Figure 8:**
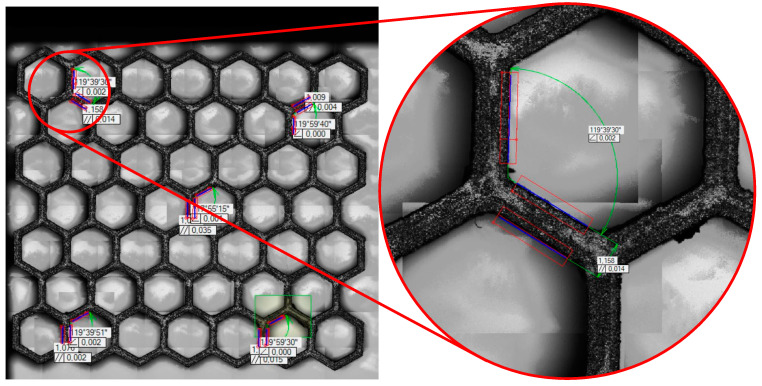
Measurements of unit cell dimensions using a Baty Venture 3030 optical coordinate measuring machine (Bradford, UK).

The cell wall thickness in the samples could have a significant influence on their energy absorption. So, the wall thickness of the 3D-printed models in the green state was scaled according to the filament manufacturer’s guidelines to achieve a final wall thickness of 1 mm after sintering. Measurements were made using a Baty Venture 3030 optical coordinate measuring machine (Bradford, UK), shown in Figure 8, using LED lighting [34]. Figure 8 shows the measurement of the internal unit cell angle and the parallelism of the wall edges. The average wall thickness and angle values for each group of samples are shown in Table 3.

### 3.2. Quasi-Static Crush Test

**Figure 9:**
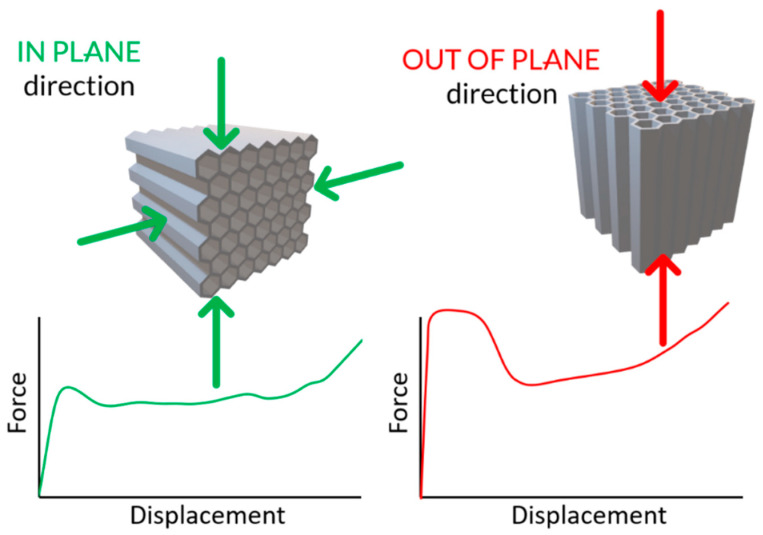
Schematic difference in the mechanical response characteristics of the regular honeycomb structure under two loading scenarios (in-plane and out-of-plane).

A schematic representation of the mechanical behavior of a conventional honeycomb structure is illustrated in Figure 9. In structural applications that involve load-bearing elements, high structural stiffness is preferred, particularly when mass reduction is crucial. For applications directed to energy absorption, the key parameter is the energy expended during deformation (represented by the area under the force–displacement history plot), along with a mechanical response, characterized by the flattest possible crushing force profile. Additionally, it is important to maximize the crushing path duration to delay the onset of full densification of the structure, thereby sustaining energy absorption over a longer period. The implementation of a zig-zag is intended to balance the magnitude and characteristics of the mechanical response in two loading directions: along the axes of the elementary cells (out-of-plane—OP) and perpendicular to these axes (in-plane—IP).

**Figure 10:**
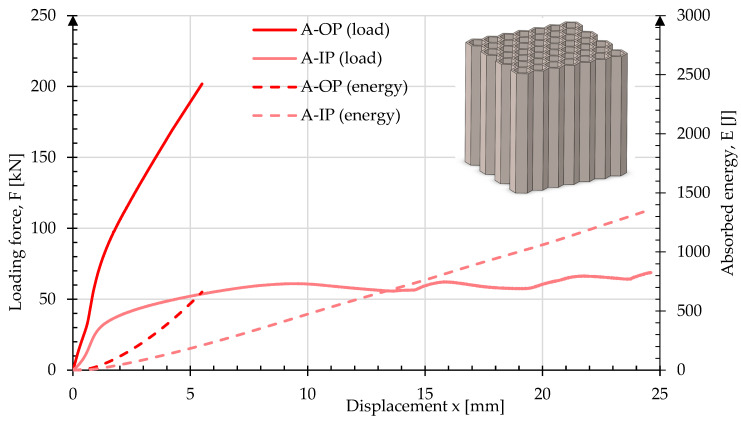
Force and energy measured during the crush of type A in both in-plane and out-of-plane loading directions.

**Figure 11:**
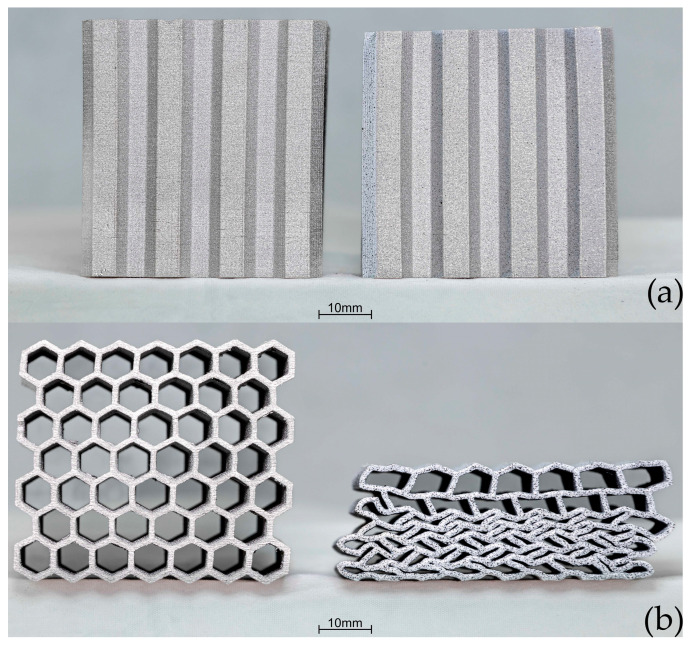
Comparison of crush results of type A before and after crush: (**a**) out-of-plane, (**b**) in-plane.

The Figure 10 shows the results of the crush test for sample A. In the OP direction, the load quickly reached a limit value of 201 kN, at 5.5 mm. The energy absorbed by the sample was 615 J. In the IP direction, the rate of change of force on reaching 34 kN at 1.62 mm displacement started to reduce suggesting the limit to elastic deformation. Then, the sample deformed by folding of the zig-zag, with a minimal increase in applied force achieving a large deformation to 25 mm, which is 50% of the initial height. The energy absorbed by the sample was 1360 J. Images of the sample geometry before and after crush are shown in Figure 11. In the OP direction, slight deformation and marginal buckling of the sample is observed. In contrast, crush in the IP direction resulted in noticeable folding of the cellular structures and a reduction in the sample’s length by about half compared to the sample before crush.

**Figure 12:**
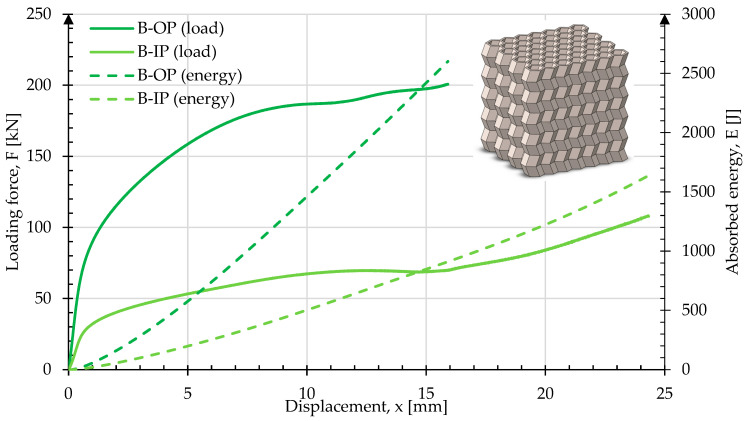
Force and energy measured during the crush of type B in both in-plane and out-of-plane loading directions.

**Figure 13:**
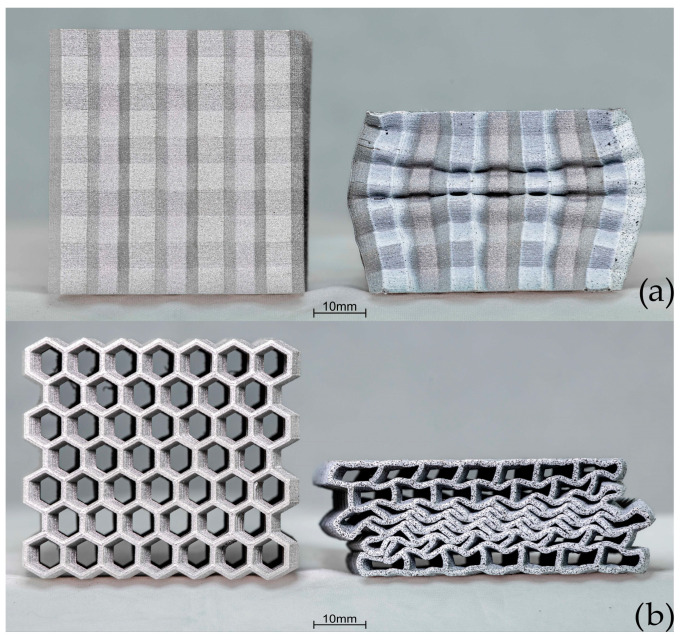
Comparison of crush results of type B before and after crush: (**a**) out-of-plane, (**b**) in-plane.

Figure 12 shows the crush test results for sample B. When comparing the curves with sample A, a notable difference is the greater deformation in sample B during crush in the OP direction. At 76 kN and 0.73 mm displacement, the rate of change in load started to reduce more quickly, reaching a maximum of 201 kN at 15.9 mm displacement. The energy absorbed by the sample was 2590 J. In the IP direction, the sample started to deform upon reaching a load of 25.1 kN. The sample reached a load of 108 kN at 24.3 mm displacement at 50% of its original height. The energy absorbed was 1640 J. Images of the sample geometry before and after crush in both the OP and IP directions are shown in Figure 13. In Figure 13b, deformation in the form of material spreading in a plane perpendicular to the force direction is visible, with two noticeable folds. For crush in the IP direction, the characteristic folding pattern is evident.

**Figure 14:**
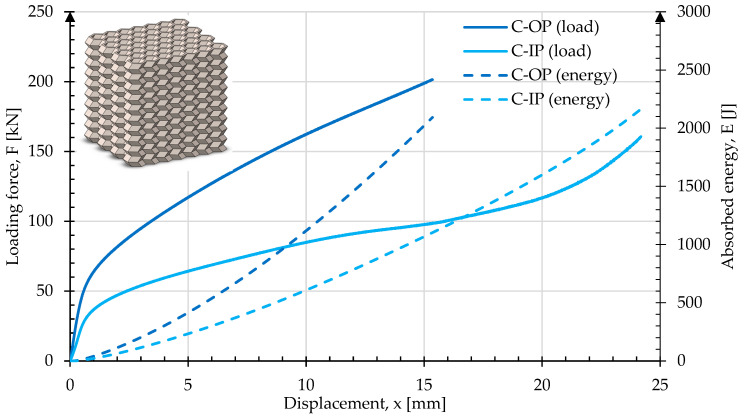
Force and energy measured during the crush of type C in both in-plane and out-of-plane loading directions.

**Figure 15:**
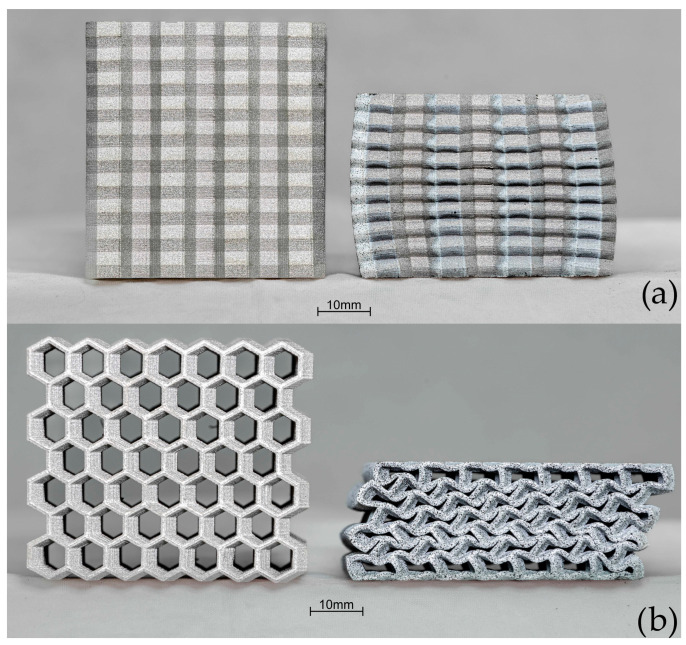
Comparison of crush results of type C before and after crush: (**a**) out-of-plane, (**b**) in-plane.

The Figure 14 shows the crush test results for sample C. In the OP direction, the rate of change in load reduces at 0.69 mm, reaching a maximum load of 201 kN and deforming by 15.3 mm. The absorbed energy absorbed was 2089 J. Compared to types A and B, the crush in the IP direction for C exhibited behavior similar to that in the OP direction. The rate of change in load at 30.8 kN and 0.66 mm displacement started to reduce. Then, the curve slope started to increase again at 98.9 kN and 15.5 mm displacement, reaching 160 kN at 50% of the original height. The energy absorbed was 2200 J. Images of the sample geometry before and after crush in both the OP and IP directions are shown in Figure 15.

**Figure 16:**
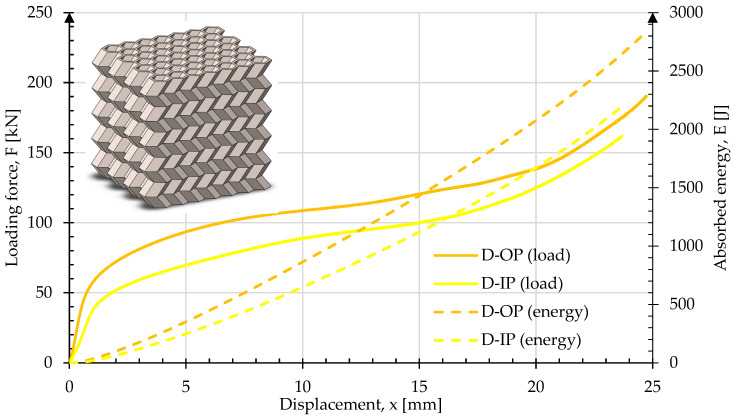
Force and energy measured during the crush of type D in both in-plane and out-of-plane loading directions.

**Figure 17:**
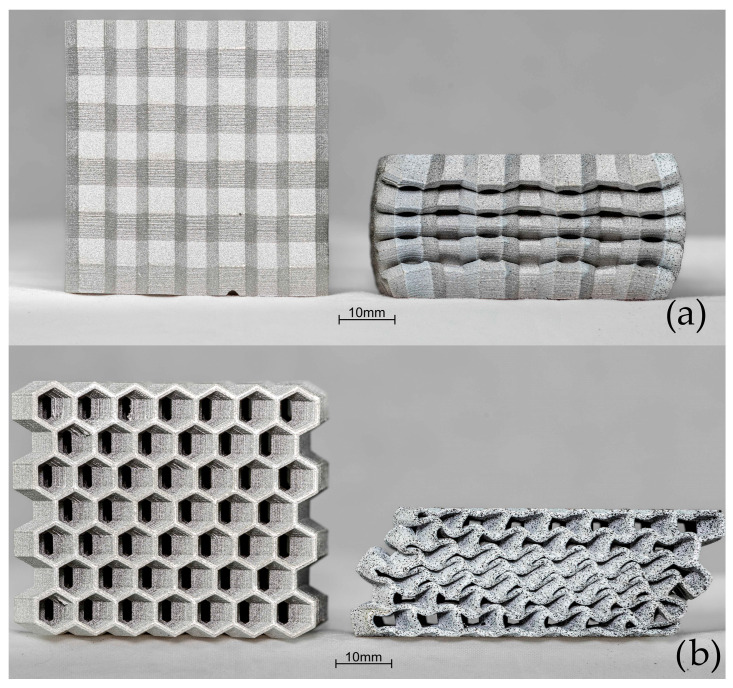
Comparison of crush results of type D before and after crush: (**a**) out-of-plane, (**b**) in-plane.

Figure 16 presents crush test results for sample D. Images of the sample geometry before and after crush in both the OP and IP directions are shown in Figure 17. In the OP direction, the rate of change in load occurred at 56.7 kN at 0.97 mm displacement. The maximum load at 50% of the original height was 198 kN. The energy absorbed was 2826 J. The curve result for crush in the IP displays a similar shape and scale to the OP direction.

**Figure 18:**
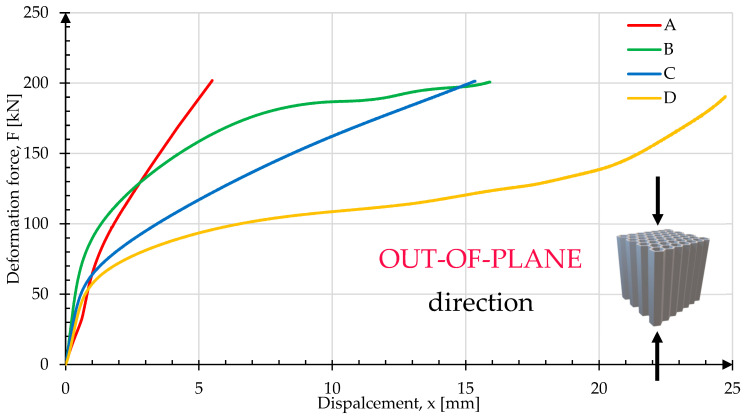
Comparison of deformation force plots registered in the out-of-plane orientation.

Figure 18 presents the results of the crush tests registered for the samples in the out-of-plane configuration. The graph shows significant differences between sample A, which exhibited the least deformation, and sample D, which compressed to half of its original length due to its specific topology properties and cell structure.

**Figure 19:**
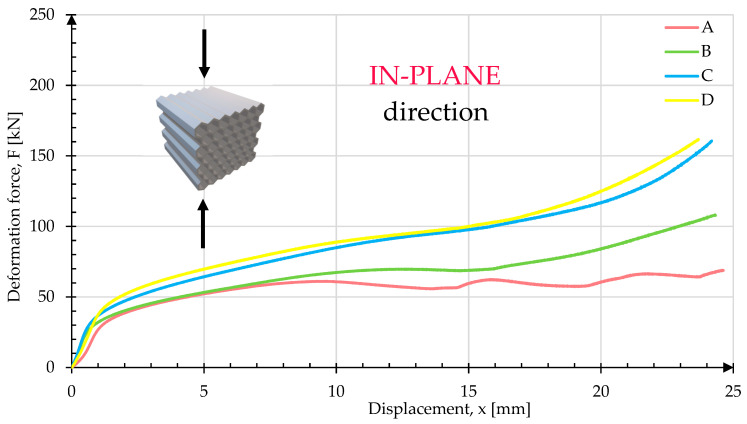
Comparison of deformation force plots registered in the in-plane orientation.

Figure 19 shows the crush test results for samples in the in-plane configuration. Fewer differences in crush behavior were observed between the different samples, as compared to the out-of-plane configuration. Nonetheless, sample A, characterized by a simple honeycomb structure, experienced the lowest compressive force, while sample D sustained the highest load.

The summary of the plastic deformation energy values for each sample was calculated and is shown in Table 4. Due to the insufficient deformation of sample A in the OP configuration, the deformation energy for this case was not considered (the result would be unreliable). The highest plastic deformation energy value of 2435 J was achieved by sample B under OP crush. The lowest plastic deformation energy value, 782 J, was recorded for sample A in IP crush. The cell topology of sample D exhibited the most similar values, with 1479 J for the OP crush and 1203 J for IP crush.

## 4. Discussion

The results presented in Figure 10, Figure 12, Figure 14, Figure 16 and Table 4 illustrate the variation in energy absorption properties of the honeycomb cell structures A, B, C and D. The introduction of a triangular zig-zag cell profile as the deformation initiator significantly reduced the peak force in the vertical loading direction (in-plane). This modification not only mitigated abrupt force increases but also made the deformation characteristic more flattened, approaching a typical plateau behavior, which is desirable for stability and energy absorption in cellular structures. The differences between the “out-of-plane” (OP) and “in-plane” (IP) crush results for samples A, B, and C highlight the different behavior of the respective lattice structures with respect to the loading direction.

For the typical honeycomb structure represented by sample A, it was observed that during crush perpendicular to the cell axes, there was a rapid and immediate folding of the individual cells. This process led to an immediate loss of structural stability, indicating that the structure was not effective in dispersing energy in this configuration. When compressed parallel to the cell axes, the structure behaved like a rigid body, with only minor barrel shape deformation, indicating a very limited energy absorption capacity in this axis. This characteristic confirms that the standard honeycomb structure has a low-energy absorption capability parallel to the cell axes, which limits its application where high-energy absorption in various loading directions is required.

In samples B and C, the introduction of geometric modifications, such as the triangular cell profile, brought noticeable improvements in the energy absorption capacity. These variants showed a gradual increase in the absorbed energy in the direction parallel to the cell axes, because of a more uniform distribution of internal stresses. This means that the modified structures, though still not ideal, demonstrated greater energy absorption capability compared to sample A, particularly in the “in-plane” direction. Such characteristics are crucial, as they allow for more controlled deformation, which enhances structural stability under dynamic loading. It is also worth noting that, despite these improvements, there was still a significant difference between the results for the OP and IP directions, indicating a need for further modifications to achieve more balanced energy absorption properties.

For sample D, a balanced energy absorption distribution in both crushing directions was observed. The load and deformation energy curves for directions parallel and perpendicular to the cell axes were very similar, indicating that sample D had comparable energy absorption properties regardless of the loading direction. This effect signifies high efficiency for this structure, making it highly desirable for applications where there is exposure to loads in various directions. The uniformity of the crush characteristics is crucial in many applications, especially where stable and predictable energy absorption is required, such as in protective elements or shock-absorbing constructions.

In summary, the results presented in this study show that modifying the geometry of the honeycomb structure has a significant impact on its energy absorption capability. The standard honeycomb structure, represented by sample A, has considerable limitations, particularly in terms of crush in OP orientation. On the other hand, samples B and C, although showing improvements, still exhibited noticeable differences dependent on the crush direction. Sample D, with its modified geometry, achieved comparable energy absorption properties in both directions, making it the most promising solution for applications where resistance to loads in various axes is crucial.

## 5. Conclusions

Based on the conducted work and an analysis of the results obtained, the following conclusions were formulated:

1. The fused filament Fabrication (FFF) technique successfully produced 316L steel cellular structure samples with consistent mass, confirming the uniformity of the manufacturing process across different structural variants. The subsequent debinding and catalytic sintering process effectively removed the binder with mass differences between variants not exceeding three grams.
2. The standard honeycomb structure (sample A) demonstrated the highest load-bearing capacity parallel to the cell axes (OP orientation), confirming its rigidity and strength in this configuration. However, limited energy absorption was observed in the OP orientation, making it less suitable for applications involving dynamic loads in various axes.
3. Variants with a triangular zig-zag profile showed reduced differences in crush characteristics in OP and IP orientations, indicating greater stability and control over the deformation process. Sample D, with a triangular deformation initiator, demonstrated the most balanced energy absorption characteristics (1479J and 1203J), making it an optimal solution for applications requiring load bearing in both OP and IP orientations.

The next step for these investigations will be to repeat the crush tests under dynamic loading conditions and perform computer simulations.

## Figures and Tables

**Table 1:** Mechanical properties of finished parts (after sintering) made of BASF Ultrafuse 316L (www.basf-3dps.com, accessed on 17 December 2024).

Print Direction	XY 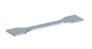	ZX 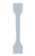
Tensile strength [MPa] (ISO 6892-1), [32]	561	521
Yield strength [MPa] (ISO 6892-1), [32]	251	234
Elongation at break [%] (ISO 6892-1), [32]	53	36
Vickers hardness (ISO 6507-1), [33]	128 HV10	128 HV10

**Table 2:** Average change in mass and dimensions of all structures topologies after the debinding and sintering processes. Green—before the process; sinter—after the process.

Type		Weight [g]	Width X [mm]	Length Y [mm]	Height Z [mm]
A	Green	274.69 (±4.95)	54.33 (±0.04)	59.76 (±0.03)	62.44 (±0.97)
	Sinter	243.24 (±4.30)	45.37 (±0.09)	50.06 (±0.08)	49.48 (±0.89)
		weight loss	shrinkage		
		31.45 (11.45%)	8.96 (16.49%)	9.70 (16.23%)	12.96 (20.76%)
B	Green	276.40 (±1.44)	54.39 (±0.06)	62.00 (±0.03)	62.90 (±0.14)
	Sinter	244.99 (±1.29)	45.48 (±0.02)	50.06 (±0.02)	49.70 (±0.10)
		weight loss	shrinkage		
		31.41 (11.36%)	8.91 (16.38%)	11.94 (19.26%)	13.20 (20.99%)
C	Green	277.18 (±0.47)	54.32 (±0.04)	61.87 (±0.10)	62.98 (±0.07)
	Sinter	245.70 (±0.42)	45.53 (±0.04)	51.82 (±0.02)	49.54 (±0.13)
		weight loss	shrinkage		
		31.48 (11.36%)	8.79 (16.18%)	10.05 (16.24%)	13.44 (21.34%)
D	Green	276.82 (±0.23)	54.37 (±0.004)	64.31 (±0.02)	62.96 (±0.06)
	Sinter	245.31 (±0.22)	45.62 (±0.05)	53.52 (±0.06)	49.06 (±0.03)
		weight loss	shrinkage		
		31.51 (11.38%)	8.75 (16.09%)	10.79 (16.78%)	13.90 (22.08%)

**Table 3:** The average wall thickness and angle values for each structure topology.

Type	Wall Thickness [mm]	Cell Angle [mm]
A	1.08 (±0.05)	119.39 (±0.86)
B	1.00 (±0.08)	119.94 (±1.25)
C	1.03 (±0.03)	122.59 (±2.54)
D	1.02 (±0.07)	122.44 (±3.15)

**Table 4:** Comparison of the plastic deformation energy values for in-plane and out-of-plane loading directions. The colors of cells correspond to the colors of the charts in the Figure 10, Figure 12, Figure 14 and Figure 16.

Topology	Out-of-Plane	In-Plane
	Plastic Deformation Energy [J]	
A	–	782
B	2435	945
C	2098	1208
D	1479	1203

## Data Availability

The original contributions presented in this study are included in the article. Further inquiries can be directed to the corresponding author.
